# Construction of a Ferroptosis-Related Nine-lncRNA Signature for Predicting Prognosis and Immune Response in Hepatocellular Carcinoma

**DOI:** 10.3389/fimmu.2021.719175

**Published:** 2021-09-17

**Authors:** Zhijie Xu, Bi Peng, Qiuju Liang, Xi Chen, Yuan Cai, Shuangshuang Zeng, Kewa Gao, Xiang Wang, Qiaoli Yi, Zhicheng Gong, Yuanliang Yan

**Affiliations:** ^1^Department of Pathology, Xiangya Hospital, Central South University, Changsha, China; ^2^Department of Pharmacy, Xiangya Hospital, Central South University, Changsha, China; ^3^National Clinical Research Center for Geriatric Disorders, Xiangya Hospital, Central South University, Changsha, China

**Keywords:** ferroptosis, immune cell infiltrate, lncRNA, hepatocellular carcinoma, survival analysis

## Abstract

Ferroptosis is an iron-dependent cell death process that plays important regulatory roles in the occurrence and development of cancers, including hepatocellular carcinoma (HCC). Moreover, the molecular events surrounding aberrantly expressed long non-coding RNAs (lncRNAs) that drive HCC initiation and progression have attracted increasing attention. However, research on ferroptosis-related lncRNA prognostic signature in patients with HCC is still lacking. In this study, the association between differentially expressed lncRNAs and ferroptosis-related genes, in 374 HCC and 50 normal hepatic samples obtained from The Cancer Genome Atlas (TCGA), was evaluated using Pearson’s test, thereby identifying 24 ferroptosis-related differentially expressed lncRNAs. The least absolute shrinkage and selection operator (LASSO) algorithm and Cox regression model were used to construct and validate a prognostic risk score model from both TCGA training dataset and GEO testing dataset (GSE40144). A nine-lncRNA-based signature (CTD-2033A16.3, CTD-2116N20.1, CTD-2510F5.4, DDX11-AS1, LINC00942, LINC01224, LINC01231, LINC01508, and ZFPM2-AS1) was identified as the ferroptosis-related prognostic model for HCC, independent of multiple clinicopathological parameters. In addition, the HCC patients were divided into high-risk and low-risk groups according to the nine-lncRNA prognostic signature. The gene set enrichment analysis enrichment analysis revealed that the lncRNA-based signature might regulate the HCC immune microenvironment by interfering with tumor necrosis factor α/nuclear factor kappa-B, interleukin 2/signal transducers and activators of transcription 5, and cytokine/cytokine receptor signaling pathways. The infiltrating immune cell subtypes, such as resting memory CD4(+) T cells, follicular helper T cells, regulatory T cells, and M0 macrophages, were all significantly different between the high-risk group and the low-risk group as indicated in Spearman’s correlation analysis. Moreover, a substantial increase in the expression of B7H3 immune checkpoint molecule was found in the high-risk group. Our findings provided a promising insight into ferroptosis-related lncRNAs in HCC and a personalized prediction tool for prognosis and immune responses in patients.

## Introduction

Hepatocellular carcinoma (HCC) is a heterogeneous tumor with increased incidence in the world (1, 2). As a common malignancy, many factors have been proven to be involved in its development, such as virus infection and cirrhosis (3). Currently, the effective treatment options for HCC predominantly include percutaneous approaches, liver transplantation, hepatic resection, *etc*. (4, 5). Even with advances in therapeutic management, the prognosis for patients with HCC remains unfavorable and poses a challenge to clinical therapists (6). Thus, uncovering novel and reliable screening methods is urgently needed to improve the diagnostic accuracy and therapeutic effect, facilitating the efforts to ameliorate the prognosis.

During the past few years, the literature suggested an increasing research progression in the area of tumor ferroptosis. Ferroptosis is a recently discovered form of reactive oxygen species (ROS)-mediated programmed cell death, which is dependent on iron metabolism and lipid peroxidation (7). The importance of ferroptosis has been demonstrated in the regulation of metabolism and redox biology, affecting the pathogenesis and treatment of cancers, including HCC. Shan and colleagues reported that ubiquitin-like modifier activating enzyme 1 promoted the development of HCC by upregulating the Nrf2 signaling pathway and downregulating Fe^2+^ levels, triggering ferroptosis inhibitory bioactivities (8). Sorafenib and sulfasalazine could synergistically inhibit the activation of branched-chain amino acid aminotransferase 2, a key enzyme participating in sulfur amino acid metabolism, resulting in ferroptosis in HCC HepG2 cells *in vitro* and *in vivo* (9). In addition, Liu et al. reported a ferroptosis- and immune-related signature and found that this prognostic signature could be used to screen the HCC patients for immunotherapies and targeted therapies (10). Therefore, understanding the underlying mechanisms and functions of ferroptosis-associated gene changes in HCC is of vital importance.

Long non-coding RNAs (lncRNAs) are non-coding transcripts of 200 nucleotides in length, which could regulate the expression of various cancer-associated genes. Recently, Zhu et al. comprehensively investigated the molecular profiles of lncRNAs in plasma samples from HCC patients and revealed that the differentially expressed lncRNAs were mainly enriched in the biological functions related to tumorigenesis, such as cell metastasis, immune response, and metabolism regulation (11). A high level of LINC00958 aggravated HCC lipogenesis and progression through sponging miR-3619-5p, further upregulating the hepatoma-derived growth factor expression (12). To date, emerging evidence have shown the potential of lncRNAs in regulating ferroptotic cell death for cancer biology. In HCC cells, a high level of lncRNA GABPB1 antisense RNA 1 enhanced erastin-induced ferroptosis by blocking GA-binding protein subunit beta-1 (GABPB1) translation and suppressing peroxiredoxin-5 peroxidase, leading to inhibition of cellular antioxidant capacity and cell viability (13). However, the application of ferroptosis–lncRNA combinations in prognostic prediction for patients with HCC remains to be elucidated.

Here a promising prognostic model for HCC was developed based on ferroptosis-associated differentially expressed lncRNAs that could be used for prognosis prediction and selection of patients for immunotherapies.

## Materials and Methods

### Data Collection

**Figure 1:**
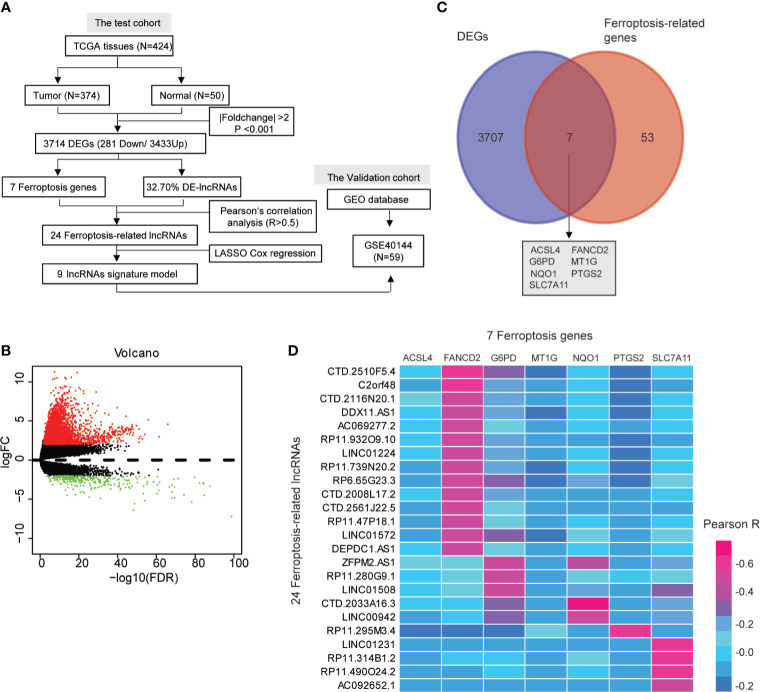
A screen of the differentially expressed ferroptosis-associated lncRNAs in hepatocellular carcinoma (HCC). **(A)** Flow chart of the analytical process in this study. **(B)** Volcano plot representing the differentially expressed genes (DEGs) between the normal and the HCC groups. The upregulated and downregulated DEGs are highlighted in red and green, respectively. **(C)** Venn diagrams of the differentially expressed ferroptosis-related genes. **(D)** Heat map showing the Pearson’s correlation between the differentially expressed-lncRNAs and the differentially expressed ferroptosis-associated genes.

Data from 424 samples, including 374 HCC tissues and 50 normal hepatic tissues, were downloaded from TCGA database up to April 1, 2021 (https://portal.gdc.cancer.gov/repository) as depicted in **Figure 1A**. The Data Transfer Tool of GDC Apps was utilized for downloading gene expression profiles and clinical information (https://tcga-data.nci.nih.gov/). The gene expression profiles were normalized using the scale method provided in the “limma” R package. Based on the set cutoff criteria of |fold-change| >2 and *P <*0.001, the differentially expressed genes (DEGs) between HCC and normal hepatic tissues were identified, and these included lncRNAs, protein-coding genes, miRNAs, *etc.* The lncRNA expression profiles and clinical information of another 59 tumor samples (GSE40144) were obtained from the GEO database (http://www.ncbi.nlm.nih.gov/geo) as testing cohort. The follow-up for the patients described in GSE40144 did not exceed 5 years.

### Identification of Ferroptosis-Related lncRNAs

To identify ferroptosis-related lncRNAs, 60 ferroptosis-related genes were retrieved from the previous literature (**Supplementary Table S1**) (14), which contains an up-to-date list of ferroptosis-related genes. Pearson’s test was performed to examine the correlation between ferroptosis-related DEGs and differentially expressed lncRNAs. Pearson’s *R* >0.5 was considered to be statistically significant.

### Cell Culture

The human embryonic hepatocyte—HHL-5—and HCC cells—MHCC97H and HUH-7—were cultured in Dulbecco’s modified Eagle’s medium, supplemented with 10% fetal bovine serum and 1% penicillin/streptomycin. The cultures were placed in a sterile incubator maintained at 37°C with 5% CO_2_. The cells in logarithmic growth phase were collected for subsequent experiments.

### RNA Extraction and Quantitative PCR

Total cellular RNA was extracted using the TRIzol reagent (Invitrogen, 15596-026) according to the protocol of the manufacturer. The cDNA synthesis was reverse-transcribed using the PrimeScript RT reagent kit (Takara, 6210, China). The qPCR assay was conducted with iTaq Universal SYBR green Supermix (Bio-Rad, 172-5850, USA). The gene expression levels of candidate lncRNAs were normalized to 18srRNA expression levels. The relative quantification of lncRNAs was calculated using the 2-ΔΔCT method (15–17). The sequences of all primers used in this study are provided in **Supplementary Table S2**.

### Apoptosis Analysis

For apoptosis analysis, HUH-7 cells were transfected with the lncRNA-targeted siRNAs (GenePharma, China). The sequences of all siRNAs used in this study are provided in **Supplementary Table S2**. Afterward, the cell apoptosis rate was analyzed using Annexin V-fluorescein isothiocyanate (BD Biosciences, USA). The Dxp AthenaTM flow cytometer (Cytek, Fremont, CA, USA) was used to analyze the results of the flow cytometry.

### Colony Forming Assay

The HUH-7 cells were transfected with lncRNA-targeted siRNAs for about 48 h. Afterward, 1,000 cells were plated in six-well culture plates and cultured for about 15 days. The cellular colonies were counted by staining with crystal violet.

### Lipid Peroxidation Assay

Lipid peroxidation was analyzed using a lipid peroxidation assay kit (Sigma, MAK085) according to the protocol of the manufacturer. Upon oxidative stress, one of the end products, such as malondialdehyde (MDA), could act as a promising marker for lipid peroxidation. Then, the reaction of MDA with thiobarbituric acid results in a pink color with a maximum absorption at 532 nm. Therefore, the levels of cellular lipid peroxidation can be identified by measuring the absorbance at 532 nm.

### Iron Assay

The concentration of ferrous (Fe^2+^) and/or ferric (Fe^3+^) iron in biological samples could be determined using an iron assay kit (Abcam, ab83366). In brief, in the acid assay buffer, the ferric carrier proteins could dissociate ferric into the solution. Then, the reaction of free ferrous iron with Ferene S results in stable-colored complexes with absorbance at 593 nm. Therefore, the levels of intracellular iron can be identified by measuring the absorbance at 593 nm.

### Construction and Validation of Ferroptosis-Related lncRNA Signature

Least absolute shrinkage and selection operator (LASSO) Cox regression of overall survival (OS) with a 10-fold cross-validation was performed to screen for ferroptosis-related lncRNAs with prognostic values. A total of 374 lncRNA-seq samples and the latest clinical follow-up information were downloaded from TCGA using GDC API (https://portal.gdc.cancer.gov/repository). Patients with unknown clinical information were excluded (*n* = 92), leaving 255 lncRNA-seq samples in the final cohort for analysis. The R package “glmnet” was used to identify the gene signature that contains the most helpful biomarkers for prognosis, and the risk score of each sample in all the datasets was calculated based on the signature (18). For the training group, the lncRNA-based prognosis risk score was established by linearly combining the following formula with the expression level-multiplied regression model (*β*): risk score = βlncRNA1 × lncRNA1 expression + βlncRNA2 × lncRNA2 expression + · ···· + βlncRNAn × lncRNAn expression. To evaluate the predictive power of the lncRNA-based prognosis risk classifier, receiver operating characteristic (ROC) of 10-year survival was analyzed using the R package “timeROC” in the training and testing datasets (19). For survival analysis, the samples were divided into high-risk group and low-risk group based on the optimal cutoff value of risk score as analyzed by the R package “survival” (20). Kaplan–Meier analysis was used to explore the prognostic significance of the ferroptosis-associated nine-lncRNA signature on HCC. Next, univariate and multivariate Cox regression analyses were conducted to assess whether this risk score model displayed good predictive ability for prognosis independent of other clinicopathological features, such as body mass index, age, gender, and pathologic staging. In addition, a prognostic nomogram was established based on the TCGA-HCC dataset. All independent prognostic parameters and relevant clinical parameters were included in the construction of a prognostic nomogram *via* a stepwise Cox regression model to predict the 3-, 5-, and 10-year OS of HCC patients in the TCGA dataset.

### Immune Infiltrate Analysis

CIBERSORT (21) was used to analyze 22 types of tumor-infiltrating immune cells (TIICs) from each sample, such as naive CD4+ T cells, CD4+ resting memory T cells, CD4+ memory-activated T cells, naive B cells, memory B cells, plasma cells, CD8+ T cells, follicular helper T cells, regulatory T cells, gammadelta T cells, M0 macrophages, M1 macrophages, M2 macrophages, resting natural killer cells, activated natural killer cells, monocytes, resting dendritic cells, activated dendritic cells, resting mast cells, activated mast cells, eosinophils, and neutrophils. The original gene expression data downloaded from TCGA was normalized prior to the CIBERSORT analysis. The statistical significance of the deconvolution results was evaluated by a derived *P*-value (*P* < 0.05) to filter out the samples with less significant accuracy. The association between the risk score of the signature and immune cells was assessed using Spearman’s correlation test. Pearson’s test was used to assess the correlation between the risk score of the signature and the expression of the immune checkpoint genes, such as programmed cell death protein 1 (PD1), PD ligand 1 (PDL1), cytotoxic T-lymphocyte-associated protein 4 (CTLA4), V-set immunoregulatory receptor (VSIR) (22), and B7H3 (23).

### Function Enrichment Analysis

Gene set enrichment analysis (GSEA) (24) was performed to identify the potential molecular mechanisms or potential functional pathways that involve the ferroptosis-related lncRNA signature. The TCGA samples were divided into a high-risk group and a low-risk group according to the optimal cutoff values. GSEA was performed in java GSEA v. 4.0.3 on the molecular signature dataset, h.all.v7.4 symbols.gmt [Hallmarks], and Kyoto Encyclopedia of Genes and Genomes (KEGG) dataset, c2.cp.kegg.v7.4.symbols.gmt, to identify enriched pathways between the high-risk group and the low-risk group. |NES| >1 and false discovery rate <0.05 were considered statistically significant.

### Statistical Analysis

All statistical analyses were performed using RStudio and its appropriate packages. *P*-values <0.05 were regarded as statistically significant.

## Results

### Identification of Differentially Expressed Ferroptosis-Associated lncRNAs

We identified 3,714 genes (3,433 upregulated and 281 downregulated) that were differentially expressed in the TCGA-HCC dataset (**Figures 1A, B** and **Supplementary Table S3**). Moreover, the pie chart exhibited that the differentially expressed lncRNAs (DE-lncRNAs) accounted for up to 32.70% of the DEGs (**Supplementary Figure S1** and **Supplementary Table S4**). Ferroptosis has been reported to be involved in the development of HCC (25, 26), so we wanted to explore whether ferroptosis-related genes existed in the DEGs. As shown in the Venn diagram (http://bioinformatics.psb.ugent.be/webtools/Venn/), seven ferroptosis-associated genes (ACSL4, FANCD2, G6PD, MT1G, NQO1, PTGS2, and SLC7A11) were identified among the 3,714 DEGs (**Figure 1C**). A total of 24 DE-lncRNAs were determined as the ferroptosis-related lncRNAs (**Supplementary Table S5**). Moreover, compared with normal hepatic tissues, 23 DE-lncRNAs were highly expressed in the HCC tissues, while only one DE-lncRNA (RP11-295M3.4) was lower in the HCC tissues (**Supplementary Table S6**). The heat map indicated a correlation between the 24 DE-lncRNAs and the seven ferroptosis-associated genes (*R* > 0.5, **Figure 1D**).

**Figure 2:**
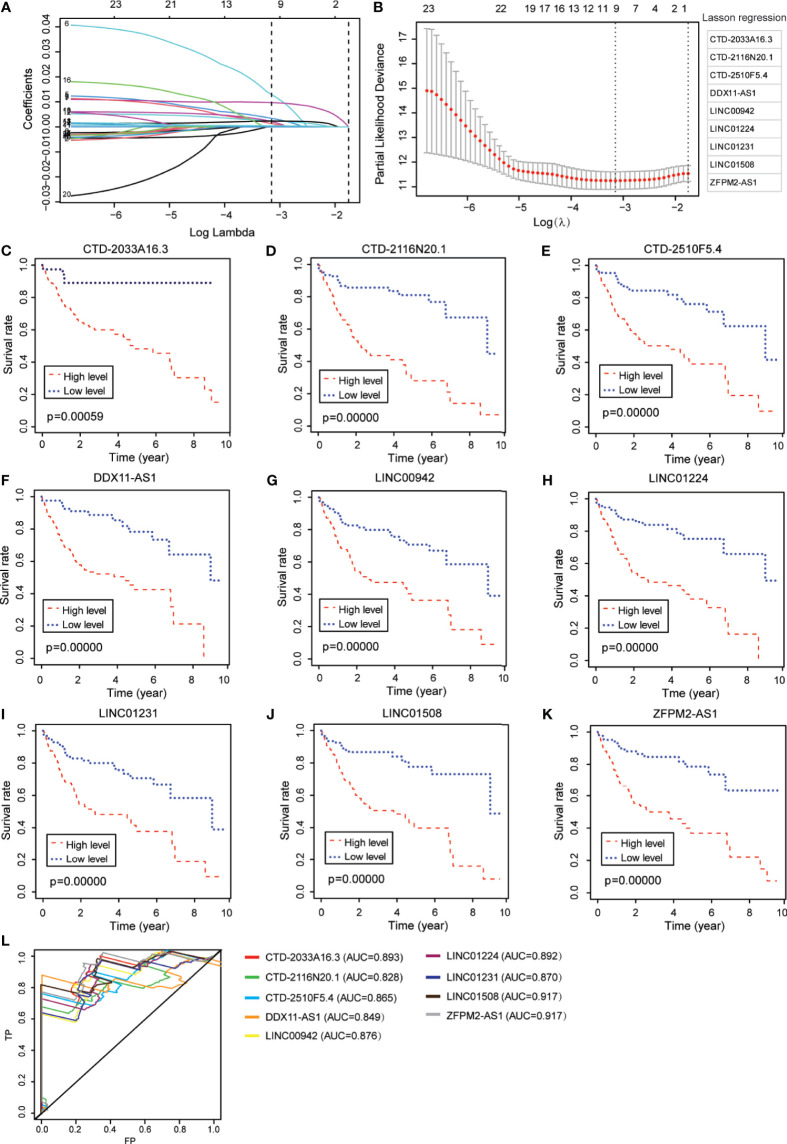
Identification of ferroptosis-associated nine-lncRNAs with prognostic value in hepatocellular carcinoma (HCC) patients. **(A, B)** LASSO Cox regression with a 10-fold cross-validation for the prognostic value of the ferroptosis-associated nine-lncRNAs, including CTD-2033A16.3, CTD-2116N20.1, CTD-2510F5.4, DDX11-AS1, LINC00942, LINC01224, LINC01231, LINC01508, and ZFPM2-AS1. **(C–K)** Kaplan–Meier analytical evaluation of the prognostic values of the candidate lncRNAs. **(L)** Time-dependent receiver operating characteristic curves for the prognostic model based on the expression of nine-lncRNAs in The Cancer Genome Atlas–HCC cohort.

**Figure 3:**
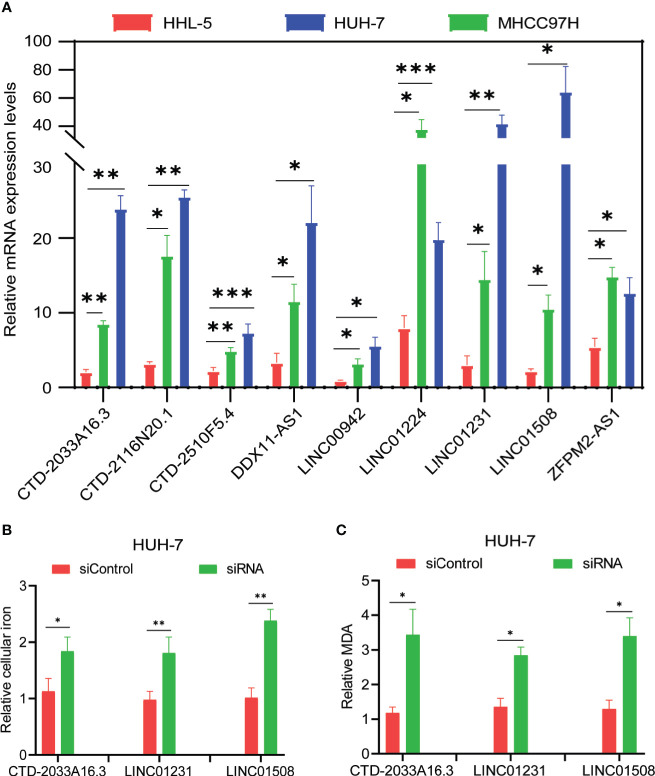
Evaluation of candidate lncRNAs in the ferroptosis of hepatocellular carcinoma cells. **(A)** The levels of candidate lncRNAs were normalized to 18srRNA. **(B)** The intracellular iron was analyzed using an iron assay kit. **(C)** The cellular malondialdehyde concentration was analyzed using a lipid peroxidation assay kit. The values are displayed as mean ± SD for three independent replicates. *p < 0.05, **p < 0.01, ***p < 0.001.

From the abovementioned 24 ferroptosis-associated lncRNAs, nine lncRNAs (CTD-2033A16.3, CTD-2116N20.1, CTD-2510F5.4, DDX11-AS1, LINC00942, LINC01224, LINC01231, LINC01508, and ZFPM2-AS1) were ultimately identified to be related to prognosis (**Figures 2A, B** and **Supplementary Table S7**). Kaplan–Meier survival analysis was further used to evaluate the significance of lncRNA expression on the prognosis of patients. As shown in **Figures 2C–K**, high levels of these candidate lncRNAs were all correlated with poor prognosis in patients with HCC. Furthermore, the time-dependent ROC analyses for the survival prediction of these key lncRNAs obtained area under the curve (AUC) values of 0.893 for CTD-2033A16.3, 0.828 for CTD-2116N20.1, 0.865 for CTD-2510F5.4, 0.849 for DDX11-AS1, 0.876 for LINC00942, 0.892 for LINC01224, 0.870 for LINC01231, 0.917 for LINC01508, and 0.917 for ZFPM2-AS1 (**Figure 2L**). In addition, qPCR showed that the expression levels of the nine candidate lncRNAs were significantly increased in two HCC cell lines—MHCC97H and HUH-7—compared to the normal liver cell line, HHL-5 (**Figure 3A**). Given that the roles of several lncRNAs, such as CTD-2033A16.3, LINC01231, and LINC01508, in HCC have not been reported, we wanted to explore whether these lncRNAs affect the apoptosis and proliferation of HCC cells. As expected, the knock-down of CTD-2033A16.3, LINC01231, and LINC01508 by siRNAs significantly promoted cell apoptosis and attenuated cell survival in the HCC cells HUH-7 (**Supplementary Figures S2A–E**). In addition, studies have indicated that intracellular iron and MDA are the characteristic features of ferroptosis (27). Next, we wanted to assess how these candidate lncRNAs regulate the cellular iron and MDA. As shown in **Figures 3B, C**, the knock-down of CTD-2033A16.3, LINC01231, and LINC01508 by siRNAs significantly improved the concentration of cellular iron and MDA in the HCC cells HUH-7, indicating their anti-ferroptosis effects. The results suggest that these ferroptosis-associated lncRNAs play important roles in HCC pathology.

### Construction of the Prognostic Signature Based on Ferroptosis-Associated Nine-lncRNAs

**Figure 4:**
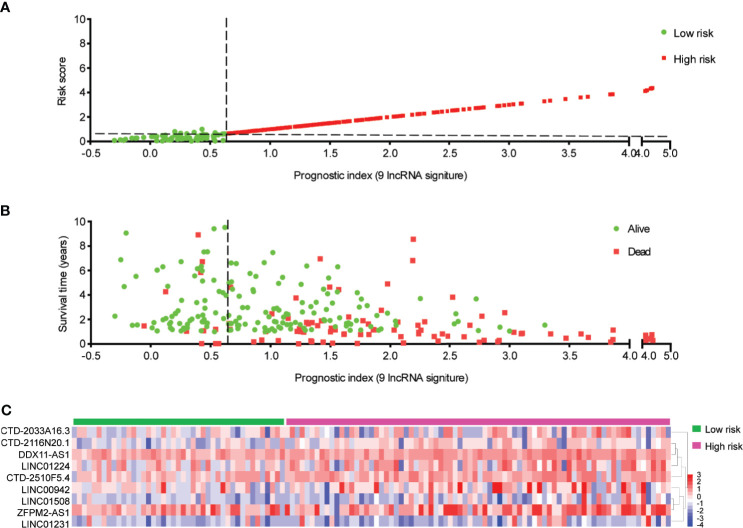
Distribution of hepatocellular carcinoma (HCC) patients based on the risk score. **(A)** Risk curve and **(B)** scatter plot for the risk score and survival status of each HCC case. The red and green dots in **(B)** represent death and survival, respectively. **(C)** Heat map showing the expression profiles of ferroptosis-associated nine-lncRNAs in the high-risk group and the low-risk group.

The nine-lncRNA expression risk score (risk score = 2.677e-05 × CTD-2033A16.3 + 1.841e-02 × CTD-2116N20.1 + 9.499e-03 × CTD-2510F5.4 + 1.016e-02 × DDX11-AS1 + 1.779e-04 × LINC00942 + 3.657e-03 × LINC01224 + 4.413e-03 × LINC01231 + 1.673e-03 × LINC01508 + 9.253e-04 × ZFPM2-AS1) for each sample was calculated (**Supplementary Table S8**). Subsequently, an X-tile diagram was used to produce the optimal cutoff point for the risk score. According to this cutoff value of risk score, the TCGA-HCC patients were divided into a high-risk group and a low-risk group. A prognostic curve and a scatter plot were used to indicate the risk score and the survival status of each HCC patient (**Figures 4A, B**). Moreover, most of the death cases were mainly distributed in the high-risk group (**Figure 4B**). In addition, the heat map of the expression profiles of candidate lncRNAs demonstrated that CTD-2033A16.3, CTD-2116N20.1, CTD-2510F5.4, DDX11-AS1, LINC00942, LINC01224, LINC01231, LINC01508, and ZFPM2-AS1 were all highly expressed in the high-risk group (**Figure 4C** and **Supplementary Table S9**). Taken together, these findings presented the ferroptosis-associated nine-lncRNAs as the prognostic signature for HCC patients.

### The Prognostic Value of Ferroptosis-Associated lncRNA Signature

**Figure 5:**
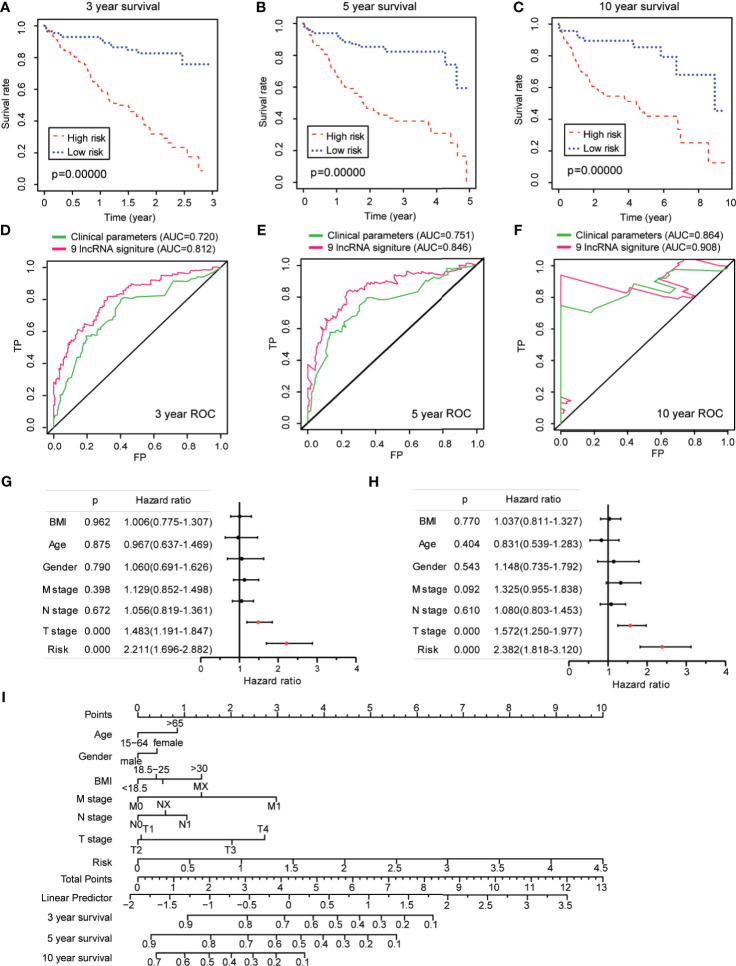
Prognostic value of ferroptosis-associated lncRNA signature in The Cancer Genome Atlas–hepatocellular carcinoma cohort. **(A–C)** Kaplan–Meier analyses for the prognostic prediction of risk score model at 3-, 5-, and 10-year survival time, respectively. **(D–F)** Time-dependent receiver operating characteristic curves for the prognostic prediction of risk score model at 3-, 5-, and 10-year survival times, respectively. **(G, H)** Univariate and multivariate Cox regression analyses for the risk score model as an independent prognostic factor. **(I)** A combined nomogram for the risk score model and other clinicopathological factors.

Kaplan–Meier analysis validated that the HCC-TCGA patients in the high-risk group displayed a significantly worse survival than those in the low-risk group for the 3-, 5-, and 10-year survival times (**Figures 5A–C**). Furthermore, the time-dependent ROC analyses showed that the AUC of the risk score model was 0.812 at 3 years, 0.846 at 5 years, and 0.908 at 10 years (**Figures 5D–F**). To demonstrate its prognostic generality, we further verified this nine-lncRNA-based risk score model with a GEO dataset (GSE40144), which contains lncRNA expression profiling and clinical survival data from 59 HCC patients. Consistent with the results from the HCC-TCGA cohort, the Kaplan–Meier curves revealed that the survival of HCC cases in the high-risk group was significantly lower than those in the low-risk group (**Supplementary Figure S3A**), and the AUC of a time-dependent ROC curve for the survival prediction of risk score model was 0.635 at 3 years (**Supplementary Figure S3B**). All these data demonstrated the superior specificity and sensitivity of this ferroptosis-associated nine-lncRNA signature than other clinical parameters.

Next, univariate Cox analysis revealed that lncRNA-based signature (hazard ratio: 2.211, 95% confidence interval: 1.696–2.882) as well as T stage (hazard ratio: 1.483, 95% confidence interval: 1.919–1.847) were independent factors for the prognosis of HCC patients (**Figure 5G**). The multivariate Cox analysis revealed likewise that both the lncRNA-based signature (hazard ratio: 2.382, 95% confidence interval: 1.818–3.120) and the T stage (hazard ratio: 1.572, 95% confidence interval: 1.250–1.977) were independent prognostic risk factors for HCC patients (**Figure 5H**). To make the lncRNA-based signature more applicable in the clinic, a nomogram was established to explore the probability of the lncRNA signature in predicting the 3-, 5-, and 10-year survival in the TCGA-HCC cohort. As shown in **Figure 5I**, the predictive factors in the nomogram contained the novel risk score model and other clinicopathological features. In this combined nomogram, the risk score model was proven to exert the most excellent weight among all these clinically relevant covariates, which was similar to the findings from the multivariable Cox regression analysis. These studies collectively verified that this novel ferroptosis-associated lncRNA signature could reliably serve as an independent prognostic factor for patients with HCC.

### GSEA Enrichment and Immunity Analysis of the Risk Score

**Figure 6:**
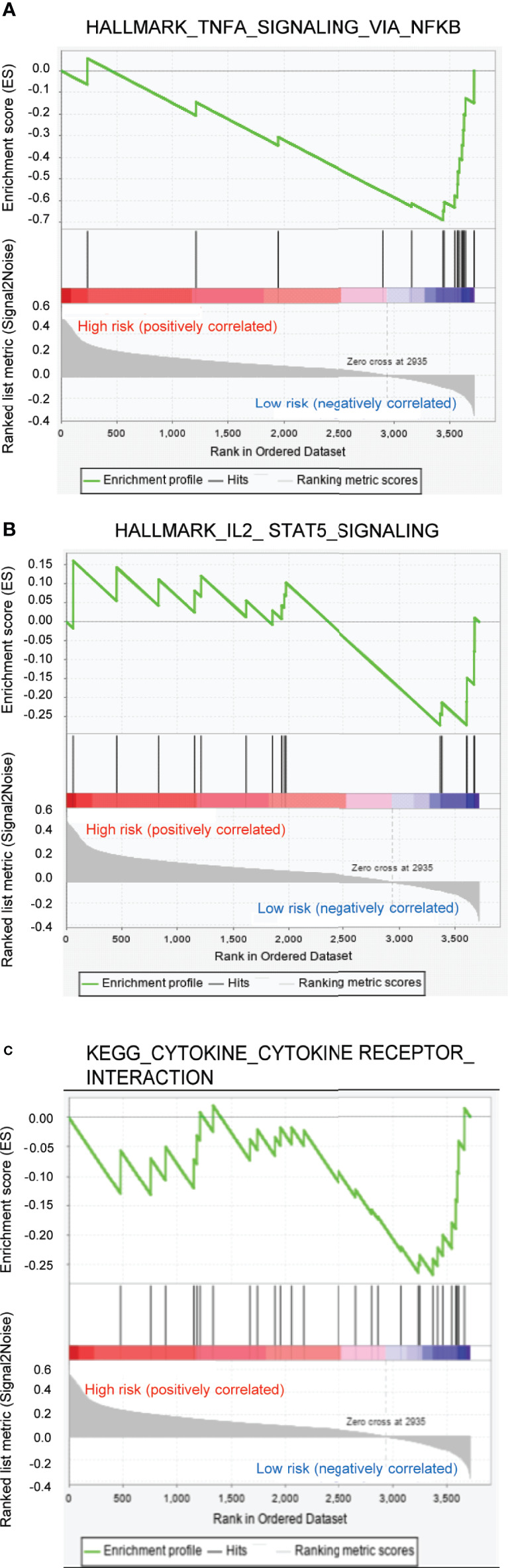
Gene set enrichment analysis enrichment analysis for ferroptosis-associated lncRNA signature. **(A, B)** Two remarkably enriched immune-associated HALLMARK pathways in low-risk patients. **(C)** Two remarkably enriched immune-associated Kyoto Encyclopedia of Genes and Genomes pathways in low-risk patients.

The GSEA analysis indicated that the tumor hallmarks correlated with the low-risk group, which may be involved in the regulation of several immune-associated signaling pathways, such as tumor necrosis factor α (TNFα)/nuclear factor kappa-B (NFκB), interleukin 2 (IL2)/signal transducers and activators of transcription 5 (STAT5), *etc.* (**Figures 6A, B**). Moreover, the GSEA analysis, along with the KEGG pathways, further revealed that the pathways correlated with the low-risk group were mainly involved in the regulation of cytokine/cytokine receptor signaling pathways (**Figure 6C**). Previous studies reported that TNFα could promote NFκB activation through binding with TNF receptor, facilitating the production of elevated levels of T-helper 1/T-helper 17-related cytokines that drive proinflammatory signaling (28). Inhibition of the TNFα/NFκB-driven proinflammatory signaling resulted in the suppression of tumor growth and progression *in vivo* and *in vitro* (29). In addition, IL2 and its downstream target STAT5 have been proven to exert an effect on multiple aspects of immune responses, for example, the regulation of T cell development and function (30, 31). Thus, GSEA enrichment confirmed the biological significance of the ferroptosis-associated lncRNA signature in immune regulation.

**Figure 7:**
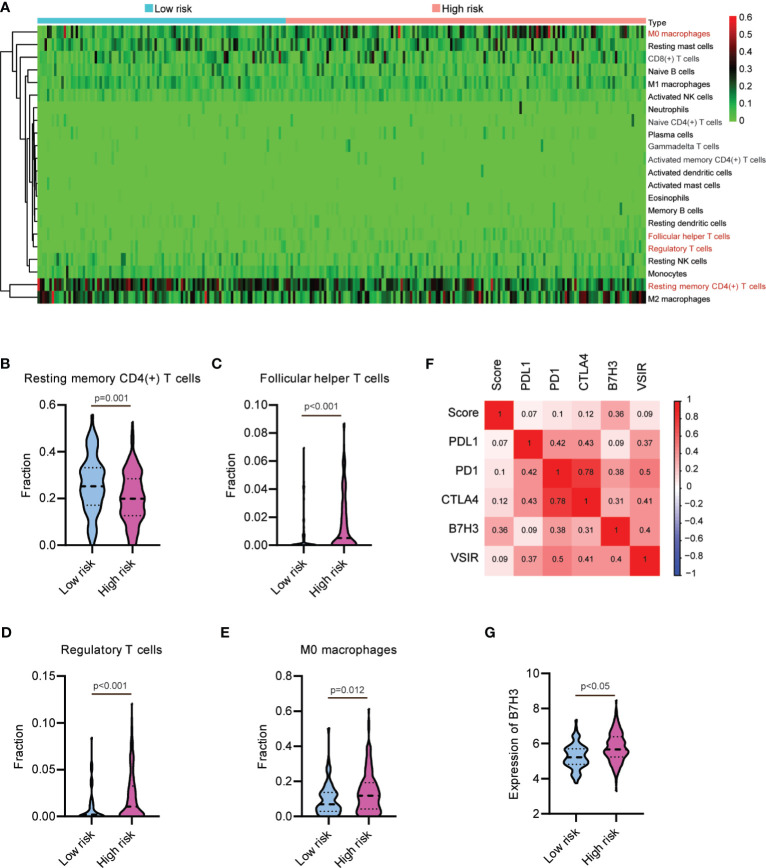
Relationship between the lncRNA-based signature and immune responses in hepatocellular carcinoma. **(A)** Heat map of immune responses based on CIBERSORT in the low-risk group and the high-risk group. The proportion of **(B)** resting memory CD4(+) T cell, **(C)** follicular helper T cells, **(D)** regulatory T cells, and **(E)** M0 macrophages in the low-risk group and the high-risk group. **(F)** Heat map showing the positive relations between risk score and several immune checkpoints. **(G)** B7H3 is upregulated in the high-risk group.

To determine whether this nine-lncRNA signature was related to tumor immunity, we next evaluated the association between the risk score and the 22 types of TIICs in HCC from the CIBERSORT algorithm (**Supplementary Figure S4**). As shown in **Figure 7A** and **Supplementary Table S10**, the heat map of immune responses based on CIBERSORT displayed that M0 macrophages and T cell functions, including follicular helper T cells, regulatory T cells, and resting memory CD4(+) T cells, were all significantly different between the high-risk group and the low-risk group. We observed significantly higher proportions of resting memory CD4(+) T cell and lower proportions of follicular helper T cells, regulatory T cells, and M0 macrophages in the high-risk group (**Figures 7B–E**). These observations implied that the infiltration of these immune cell subtypes might exert an important influence on the prognosis of HCC patients. Given the clinical importance of therapeutic strategies based on immune checkpoint blockade in HCC (32, 33), we then explore the association between the risk score and several immune checkpoints, such as PD1, PDL1, CTLA4, VSIR, and B7H3. As shown in **Figure 7F**, the heat map showed the positive relations between risk score and these immune checkpoints. Moreover, a substantial increase in the expression of B7H3 was found in the high-risk group (**Figure 7G**). Taken together, these data suggested that the ferroptosis-associated lncRNA signature might affect the response to immunotherapy in HCC patients.

## Discussion

HCC is one of the most common malignancies with a high mortality in the world. Identifying reliable and effective biomarkers for HCC prognosis is of great importance. Here we identified a novel ferroptosis-related nine-lncRNA signature in a large-scale HCC cohort, including a testing dataset and a validation dataset, demonstrating its sensitivity and specificity.

In previous investigations, the lncRNA signatures for prognostic prediction have been described in many kinds of cancers, such as breast cancer (34), gastric cancer (35), *etc*. Similarly, based on the differentially expressed lncRNAs and disease pathogenesis, several lncRNA-associated signatures have also been developed to predict the outcome of HCC patients. A six-lncRNA signature (MSC-AS1, POLR2J4, EIF3J-AS1, SERHL, RMST, and PVT1) could be used to effectively predict the HCC recurrence risk (36). Another autophagy-related four-lncRNA signature (LUCAT1, AC099850.3, ZFPM2-AS1, and AC009005.1) has been developed to evaluate the autophagy-related regulatory mechanisms of identified lncRNAs in HCC outcome (37). However, the ferroptosis–lncRNA interaction in the HCC prognostic model remains to be clarified. Here we report for the first time the ferroptosis-related lncRNA signature for prognosis and immune response of HCC populations, providing a promising strategy with an important clinical implication for guiding individual therapy and improving outcome prediction. In addition, the biological functions of candidate lncRNAs in the pathology of human cancers have been proven in several independent reports, for example, the high-level expression of CTD-2510F5.4 strengthened the malignant phenotype in gastric cancer (38). Knockdown of DDX11-AS1 significantly inhibited cell proliferation and migration in HCC *in vitro* and *in vivo* (39). LINC01224 silently repressed the HCC progression through sponging of microRNA-330-5p (40).

To date, ferroptosis has been recognized as a form of regulated cell death (41), which displays functional roles in HCC tumorigenesis and immune regulation. A newly identified circular RNA, Circ0097009, could upregulate the expression of SLC7A11, a key ferroptosis-associated regulator, by sponging miR-1261 in multiple HCC cell lines (42). O-GlcNAcylation-mediated YAP stabilization enhanced the sensitivity of HCC cells to RSL3-induced ferroptosis *in vitro* and *in vivo* (43). Recent studies have reported the direct crosstalk between ferroptosis and anti-tumor immunity. Tumor-infiltrating lymphocyte-mediated ferroptosis can effectively enhance the efficacy of immune checkpoint inhibitors. Iron overload in cancer cells is considered to boost the immune checkpoint blockade in HCC therapy *via* stimulating ROS accumulation and sensitizing cancer cells to ferroptosis (44, 45). Thus, explorations focusing on the detailed mechanisms and functions of ferroptosis in HCC will help pave the way to identify ferroptosis induction as a promising therapeutic method.

Through strengthening the immune system of patients, immunotherapy has been shown to be successful in making cancer a curable disease in various malignancies. A considerable body of preclinical and clinical literatures highlight that immune-based therapeutic strategies offer survival benefits for HCC. Moreover, a combination of immunotherapy and other therapeutic methods is likely to become an alternative option in HCC treatment (46, 47). Wen et al. synthetized a double-stranded polyinosinic-polycytidylic acid (polyIC) and demonstrated that the combination of polyIC with checkpoint inhibitors could distinctly activate the anti-tumor immune, thus effectively preventing liver tumorigenesis (48). In addition, emerging studies have proven the importance of tumor-infiltrating lymphocytes in driving immune evasion during HCC progression, including regulatory T cells, tumor-associated macrophages, *etc.* (49). The exhaustion of follicular helper T cells induced by intra-tumoral PDL1 resulted in the defective B cell function, facilitating the progression of advanced HCC (50). In this study, high levels of follicular helper T cells, regulatory T cells, and M0 macrophages were found in the high-risk group, indicating immune tolerance in the high-risk HCC patients. Thus, the ferroptosis-related lncRNA signature could provide potential cues for the patient selection for more effective anti-tumor immunotherapies. However, additional validation is required to understand the roles of our signature in the prediction of immunotherapeutic response in HCC patients.

However, there are several limitations in our study. Our report is mainly based on integrative bioinformatics, and effective experimental validation for these findings is currently lacking. Furthermore, the accuracy of ferroptosis-related lncRNA signature for the prognosis and immune regulation of HCC patients will remain an important issue in the clinic. In particular, the guidelines for the clinical use of this prognostic risk score model needs to be further defined.

## Conclusion

In conclusion, a novel ferroptosis-related nine-lncRNA signature was constructed as an efficient computational technique for predicting the prognosis and immune response of patients with HCC. This signature was robustly connected to the risk scores, survival time, and tumor clinical parameters. Immune analysis supported the association between the risk value of this signature and specific immune cell populations. Thus, our findings suggested a promising insight into ferroptosis-related lncRNAs in the HCC population and provided a personalized prediction tool for prognosis and immune responses.

## Data Availability Statement

The original contributions presented in the study are included in the article/**Supplementary Material**. Further inquiries can be directed to the corresponding author.

## Author Contributions

ZX, ZG, and YY contributed to the conception and design of the study. BP, QL, XC, YC, and SZ contributed to the writing, review, and/or revision of the manuscript. KG, XW, and QY provided administrative, technical, or material support. All authors contributed to the article and approved the submitted version.

## Funding

This study is supported by grants from the China Postdoctoral Science Foundation (grant numbers 2021T140754 and 2020M672521), the National Natural Science Foundation of China (grant number 81803035), the Natural Science Foundation of Hunan Province (grant numbers 2019JJ50932 and 2020JJ5934), and the Postdoctoral Science Foundation of Central South University (grant number 248485).

## Conflict of Interest

The authors declare that the research was conducted in the absence of any commercial or financial relationships that could be construed as a potential conflict of interest.

## Publisher’s Note

All claims expressed in this article are solely those of the authors and do not necessarily represent those of their affiliated organizations, or those of the publisher, the editors and the reviewers. Any product that may be evaluated in this article, or claim that may be made by its manufacturer, is not guaranteed or endorsed by the publisher.

## Supplementary Material

Supplementary Figure 1 Pie chart for the composition of differentially expressed genes (DEGs). The DEGs consisted of 53.28% protein-coding genes, 32.70% lncRNAs, 0.44% miRNAs, *etc.*

Supplementary Figure 2 Effects of CTD-2033A16.3, LINC01231, and LINC01508 on the apoptosis and proliferation of hepatocellular carcinoma (HCC) cells HUH-7. **(A)** qPCR analysis of lncRNA expression after transfection with the lncRNA-targeted siRNAs. **(B, C)** The inhibition of CTD-2033A16.3, LINC01231, and LINC01508 by siRNAs leads to the promotion of cell apoptosis rate. **(D, E)** The inhibition of CTD-2033A16.3, LINC01231, and LINC01508 by siRNAs leads to the inhibition of the cell colony forming rate. Values are displayed as mean ± SD for three independent replicates. **p* < 0.05, ***p* < 0.01.

Supplementary Figure 3 Prognostic value of ferroptosis-associated lncRNA signature in GSE40144. **(A)** Kaplan–Meier analysis was used to verify the prognostic values of novel lncRNA-based signature in hepatocellular carcinoma patients from GSE40144. **(B)** Time-dependent receiver operating characteristic curve for the survival prediction of the risk score model in the GSE40144 cohort.

Supplementary Figure 4 Spearman’s correlation on the association between the risk score of the signature and immune cells.

## References

[1] Liu P Tang Q Chen M Chen W Lu Y Liu Z. Hepatocellular Senescence: Immunosurveillance and Future Senescence-Induced Therapy in Hepatocellular Carcinoma. Front Oncol (2020) 10:589908. doi: 10.3389/fonc.2020.589908 33330071 PMC7732623

[2] Huang X Qin F Meng Q Dong M. Protein Tyrosine Phosphatase Receptor Type D (PTPRD)-Mediated Signaling Pathways for the Potential Treatment of Hepatocellular Carcinoma: A Narrative Review. Ann Trans Med (2020) 8(18):1192. doi: 10.21037/atm-20-4733 PMC7576031 33241041

[3] Cheng CW Tse E. Targeting PIN1 as a Therapeutic Approach for Hepatocellular Carcinoma. Front Cell Dev Biol (2019) 7:369. doi: 10.3389/fcell.2019.00369 32010690 PMC6974617

[4] Li J Zhu Y. Recent Advances in Liver Cancer Stem Cells: Non-Coding RNAs, Oncogenes and Oncoproteins. Front Cell Dev Biol (2020) 8:548335. doi: 10.3389/fcell.2020.548335 33117795 PMC7575754

[5] Feng D Wang M Hu J Li S Zhao S Li H. Prognostic Value of the Albumin-Bilirubin Grade in Patients With Hepatocellular Carcinoma and Other Liver Diseases. Ann Trans Med (2020) 8(8):553. doi: 10.21037/atm.2020.02.116 PMC7214886 32411776

[6] Li W Chen QF Huang T Wu P Shen L Huang ZL. Identification and Validation of a Prognostic lncRNA Signature for Hepatocellular Carcinoma. Front Oncol (2020) 10:780. doi: 10.3389/fonc.2020.00780 32587825 PMC7298074

[7] Tang R Hua J Xu J Liang C Meng Q Liu J. The Role of Ferroptosis Regulators in the Prognosis, Immune Activity and Gemcitabine Resistance of Pancreatic Cancer. Ann Trans Med (2020) 8(21):1347. doi: 10.21037/atm-20-2554a PMC7723621 33313092

[8] Shan Y Yang G Huang H Zhou Y Hu X Lu Q. Ubiquitin-Like Modifier Activating Enzyme 1 as a Novel Diagnostic and Prognostic Indicator That Correlates With Ferroptosis and the Malignant Phenotypes of Liver Cancer Cells. Front Oncol (2020) 10:592413. doi: 10.3389/fonc.2020.592413 33344241 PMC7744729

[9] Wang K Zhang Z Tsai HI Liu Y Gao J Wang M. Branched-Chain Amino Acid Aminotransferase 2 Regulates Ferroptotic Cell Death in Cancer Cells. Cell Death Differ (2021) 28(4):1222–36. doi: 10.1038/s41418-020-00644-4 PMC8027606 33097833

[10] Liu Y Zhang X Zhang J Tan J Li J Song Z. Development and Validation of a Combined Ferroptosis and Immune Prognostic Classifier for Hepatocellular Carcinoma. Front Cell Dev Biol (2020) 8:596679. doi: 10.3389/fcell.2020.596679 33425905 PMC7785857

[11] Zhu Y Wang S Xi X Zhang M Liu X Tang W. Integrative Analysis of Long Extracellular RNAs Reveals a Detection Panel of Noncoding RNAs for Liver Cancer. Theranostics (2021) 11(1):181–93. doi: 10.7150/thno.48206 PMC7681086 33391469

[12] Zuo X Chen Z Gao W Zhang Y Wang J Wang J. M6A-Mediated Upregulation of LINC00958 Increases Lipogenesis and Acts as a Nanotherapeutic Target in Hepatocellular Carcinoma. J Hematol Oncol (2020) 13(1):5. doi: 10.1186/s13045-019-0839-x 31915027 PMC6951025

[13] Qi W Li Z Xia L Dai J Zhang Q Wu C. LncRNA GABPB1-AS1 and GABPB1 Regulate Oxidative Stress During Erastin-Induced Ferroptosis in HepG2 Hepatocellular Carcinoma Cells. Sci Rep (2019) 9(1):16185. doi: 10.1038/s41598-019-52837-8 31700067 PMC6838315

[14] Liang JY Wang DS Lin HC Chen XX Yang H Zheng Y. A Novel Ferroptosis-Related Gene Signature for Overall Survival Prediction in Patients With Hepatocellular Carcinoma. Int J Biol Sci (2020) 16(13):2430–41. doi: 10.7150/ijbs.45050 PMC7378635 32760210

[15] Pandelides Z Thornton C Faruque AS Whitehead AP Willett KL Ashpole NM. Developmental Exposure to Cannabidiol (CBD) Alters Longevity and Health Span of Zebrafish (Danio Rerio). GeroScience (2020) 42(2):785–800. doi: 10.1007/s11357-020-00182-4 32221778 PMC7205952

[16] Buford TW Sun Y Roberts LM Banerjee A Peramsetty S Knighton A. Angiotensin (1-7) Delivered Orally *via* Probiotic, But Not Subcutaneously, Benefits the Gut-Brain Axis in Older Rats. GeroScience (2020) 42(5):1307–21. doi: 10.1007/s11357-020-00196-y PMC7525634 32451847

[17] Kiss T Giles CB Tarantini S Yabluchanskiy A Balasubramanian P Gautam T. Nicotinamide Mononucleotide (NMN) Supplementation Promotes Anti-Aging miRNA Expression Profile in the Aorta of Aged Mice, Predicting Epigenetic Rejuvenation and Anti-Atherogenic Effects. GeroScience (2019) 41(4):419–39. doi: 10.1007/s11357-019-00095-x PMC6815288 31463647

[18] Friedman J Hastie T Tibshirani R. Regularization Paths for Generalized Linear Models *via* Coordinate Descent. J Stat Softw (2010) 33(1):1–22. 20808728 PMC2929880

[19] Blanche P Dartigues JF Jacqmin-Gadda H. Estimating and Comparing Time-Dependent Areas Under Receiver Operating Characteristic Curves for Censored Event Times With Competing Risks. Stat Med (2013) 32(30):5381–97. doi: 10.1002/sim.5958 24027076

[20] Eaton A Therneau T Le-Rademacher J. Designing Clinical Trials With (Restricted) Mean Survival Time Endpoint: Practical Considerations. Clin Trials (2020) 17(3):285–94. doi: 10.1177/1740774520905563 32063031

[21] Newman AM Liu CL Green MR Gentles AJ Feng W Xu Y. Robust Enumeration of Cell Subsets From Tissue Expression Profiles. Nat Methods (2015) 12(5):453–7. doi: 10.1038/nmeth.3337 PMC4739640 25822800

[22] Pilones KA Hensler M Daviaud C Kraynak J Fucikova J Galluzzi L. Converging Focal Radiation and Immunotherapy in a Preclinical Model of Triple Negative Breast Cancer: Contribution of VISTA Blockade. Oncoimmunology (2020) 9(1):1830524. doi: 10.1080/2162402X.2020.1830524 33150045 PMC7583495

[23] Picarda E Ohaegbulam KC Zang X. Molecular Pathways: Targeting B7-H3 (CD276) for Human Cancer Immunotherapy. Clin Cancer Res (2016) 22(14):3425–31. doi: 10.1158/1078-0432.CCR-15-2428 PMC4947428 27208063

[24] Subramanian A Tamayo P Mootha VK Mukherjee S Ebert BL Gillette MA. Gene Set Enrichment Analysis: A Knowledge-Based Approach for Interpreting Genome-Wide Expression Profiles. Proc Natl Acad Sci USA (2005) 102(43):15545–50. doi: 10.1073/pnas.0506580102 PMC1239896 16199517

[25] Jin M Shi C Li T Wu Y Hu C Huang G. Solasonine Promotes Ferroptosis of Hepatoma Carcinoma Cells *via* Glutathione Peroxidase 4-Induced Destruction of the Glutathione Redox System. Biomed Pharmacother = Biomed Pharmacother (2020) 129:110282. doi: 10.1016/j.biopha.2020.110282 32531676

[26] Sun J Zhou C Zhao Y Zhang X Chen W Zhou Q. Quiescin Sulfhydryl Oxidase 1 Promotes Sorafenib-Induced Ferroptosis in Hepatocellular Carcinoma by Driving EGFR Endosomal Trafficking and Inhibiting NRF2 Activation. Redox Biol (2021) 41:101942. doi: 10.1016/j.redox.2021.101942 33770521 PMC8024711

[27] Mancuso RI Foglio MA Olalla Saad ST. Artemisinin-Type Drugs for the Treatment of Hematological Malignancies. Cancer Chemother Pharmacol (2021) 87(1):1–22. doi: 10.1007/s00280-020-04170-5 33141328

[28] Lin WJ Su YW Lu YC Hao Z Chio II Chen NJ. Crucial Role for TNF Receptor-Associated Factor 2 (TRAF2) in Regulating NFkappaB2 Signaling That Contributes to Autoimmunity. Proc Natl Acad Sci USA (2011) 108(45):18354–9. doi: 10.1073/pnas.1109427108 PMC3215017 22042853

[29] Pollard BS Suckow MA Wolter WR Starr JM Eidelman O Dalgard CL. Digitoxin Inhibits Epithelial-To-Mesenchymal-Transition in Hereditary Castration Resistant Prostate Cancer. Front Oncol (2019) 9:630. doi: 10.3389/fonc.2019.00630 31428571 PMC6687970

[30] Czerwinska P Rucinski M Wlodarczyk N Jaworska A Grzadzielewska I Gryska K. Therapeutic Melanoma Vaccine With Cancer Stem Cell Phenotype Represses Exhaustion and Maintains Antigen-Specific T Cell Stemness by Up-Regulating BCL6. Oncoimmunology (2020) 9(1):1710063. doi: 10.1080/2162402X.2019.1710063 32002306 PMC6959432

[31] Mahmud SA Manlove LS Farrar MA. Interleukin-2 and STAT5 in Regulatory T Cell Development and Function. Jak-Stat (2013) 2(1):e23154. doi: 10.4161/jkst.23154 24058794 PMC3670270

[32] Wing-Sum Cheu J Chak-Lui Wong C. Mechanistic Rationales Guiding Combination Hepatocellular Carcinoma Therapies Involving Immune Checkpoint Inhibitors. Hepatology (2021). doi: 10.1002/hep.31840 33811765

[33] Yang W Feng Y Zhou J Cheung OK Cao J Wang J. A Selective HDAC8 Inhibitor Potentiates Antitumor Immunity and Efficacy of Immune Checkpoint Blockade in Hepatocellular Carcinoma. Sci Trans Med (2021) 13(588):eaaz6804. doi: 10.1126/scitranslmed.aaz6804 33827976

[34] Sun M Liu X Xia L Chen Y Kuang L Gu X. A nine-lncRNA Signature Predicts Distant Relapse-Free Survival of HER2-Negative Breast Cancer Patients Receiving Taxane and Anthracycline-Based Neoadjuvant Chemotherapy. Biochem Pharmacol (2020) 189:114285. doi: 10.1016/j.bcp.2020.114285 33069665

[35] He Y Wang X. Identification of Molecular Features Correlating With Tumor Immunity in Gastric Cancer by Multi-Omics Data Analysis. Ann Trans Med (2020) 8(17):1050. doi: 10.21037/atm-20-922 PMC7575957 33145269

[36] Gu JX Zhang X Miao RC Xiang XH Fu YN Zhang JY. Six-Long non-Coding RNA Signature Predicts Recurrence-Free Survival in Hepatocellular Carcinoma. World J Gastroenterol (2019) 25(2):220–32. doi: 10.3748/wjg.v25.i2.220 PMC6337021 30670911

[37] Zhang Y Zhang L Xu Y Wu X Zhou Y Mo J. Immune-Related Long Noncoding RNA Signature for Predicting Survival and Immune Checkpoint Blockade in Hepatocellular Carcinoma. J Cell Physiol (2020) 235(12):9304–16. doi: 10.1002/jcp.29730 32330311

[38] Wang Z Qin B. Prognostic and Clinicopathological Significance of Long Noncoding RNA CTD-2510F5.4 in Gastric Cancer. Gastric Cancer (2019) 22(4):692–704. doi: 10.1007/s10120-018-00911-x 30560474 PMC6570689

[39] Li Y Zhuang W Huang M Li X. Long Noncoding RNA DDX11-AS1 Epigenetically Represses LATS2 by Interacting With EZH2 and DNMT1 in Hepatocellular Carcinoma. Biochem Biophys Res Commun (2019) 514(4):1051–7. doi: 10.1016/j.bbrc.2019.05.042 31097223

[40] Gong D Feng PC Ke XF Kuang HL Pan LL Ye Q. Silencing Long Non-Coding RNA LINC01224 Inhibits Hepatocellular Carcinoma Progression *via* MicroRNA-330-5p-Induced Inhibition of CHEK1. Mol Ther Nucleic Acids (2020) 19:482–97. doi: 10.1016/j.omtn.2019.10.007 PMC6948252 31902747

[41] Wei R Qiu H Xu J Mo J Liu Y Gui Y. Expression and Prognostic Potential of GPX1 in Human Cancers Based on Data Mining. Ann Trans Med (2020) 8(4):124. doi: 10.21037/atm.2020.02.36 PMC7049064 32175417

[42] Lyu N Zeng Y Kong Y Chen Q Deng H Ou S. Ferroptosis Is Involved in the Progression of Hepatocellular Carcinoma Through the Circ0097009/miR-1261/SLC7A11 Axis. Ann Trans Med (2021) 9(8):675. doi: 10.21037/atm-21-997 PMC8106082 33987373

[43] Zhu G Murshed A Li H Ma J Zhen N Ding M. O-GlcNAcylation Enhances Sensitivity to RSL3-Induced Ferroptosis *via* the YAP/TFRC Pathway in Liver Cancer. Cell Death Discov (2021) 7(1):83. doi: 10.1038/s41420-021-00468-2 33863873 PMC8052337

[44] Zhang W Wang F Hu C Zhou Y Gao H Hu J. The Progress and Perspective of Nanoparticle-Enabled Tumor Metastasis Treatment. Acta Pharm Sin B (2020) 10(11):2037–53. doi: 10.1016/j.apsb.2020.07.013 PMC7714986 33304778

[45] Alu A Han X Ma X Wu M Wei Y Wei X. The Role of Lysosome in Regulated Necrosis. Acta Pharm Sin B (2020) 10(10):1880–903. doi: 10.1016/j.apsb.2020.07.003 PMC7606114 33163342

[46] Obi S Sato T Sato S. Immune Checkpoint Inhibitor in Liver Cancer-Unique Regional Differences. Ann Trans Med (2020) 8(21):1336. doi: 10.21037/atm-20-3378 PMC7723575 33313081

[47] Tang X Shu Z Zhang W Cheng L Yu J Zhang M. Clinical Significance of the Immune Cell Landscape in Hepatocellular Carcinoma Patients With Different Degrees of Fibrosis. Ann Trans Med (2019) 7(20):528. doi: 10.21037/atm.2019.09.122 PMC6861814 31807510

[48] Wen L Xin B Wu P Lin CH Peng C Wang G. An Efficient Combination Immunotherapy for Primary Liver Cancer by Harmonized Activation of Innate and Adaptive Immunity in Mice. Hepatology (2019) 69(6):2518–32. doi: 10.1002/hep.30528 PMC6541536 30693544

[49] Lurje I Werner W Mohr R Roderburg C Tacke F Hammerich L. *In Situ* Vaccination as a Strategy to Modulate the Immune Microenvironment of Hepatocellular Carcinoma. Front Immunol (2021) 12:650486. doi: 10.3389/fimmu.2021.650486 34025657 PMC8137829

[50] Zhou ZQ Tong DN Guan J Tan HW Zhao LD Zhu Y. Follicular Helper T Cell Exhaustion Induced by PD-L1 Expression in Hepatocellular Carcinoma Results in Impaired Cytokine Expression and B Cell Help, and Is Associated With Advanced Tumor Stages. Am J Trans Res (2016) 8(7):2926–36. PMC4969429 27508013

