# Supplementary figures and images for: Construction of a Ferroptosis-Related Nine-lncRNA Signature for Predicting Prognosis and Immune Response in Hepatocellular Carcinoma
Source: Front Immunol. 2021 Sep 17;12:719175. doi: 10.3389/fimmu.2021.719175 (PMC8484522; doi:10.3389/fimmu.2021.719175)

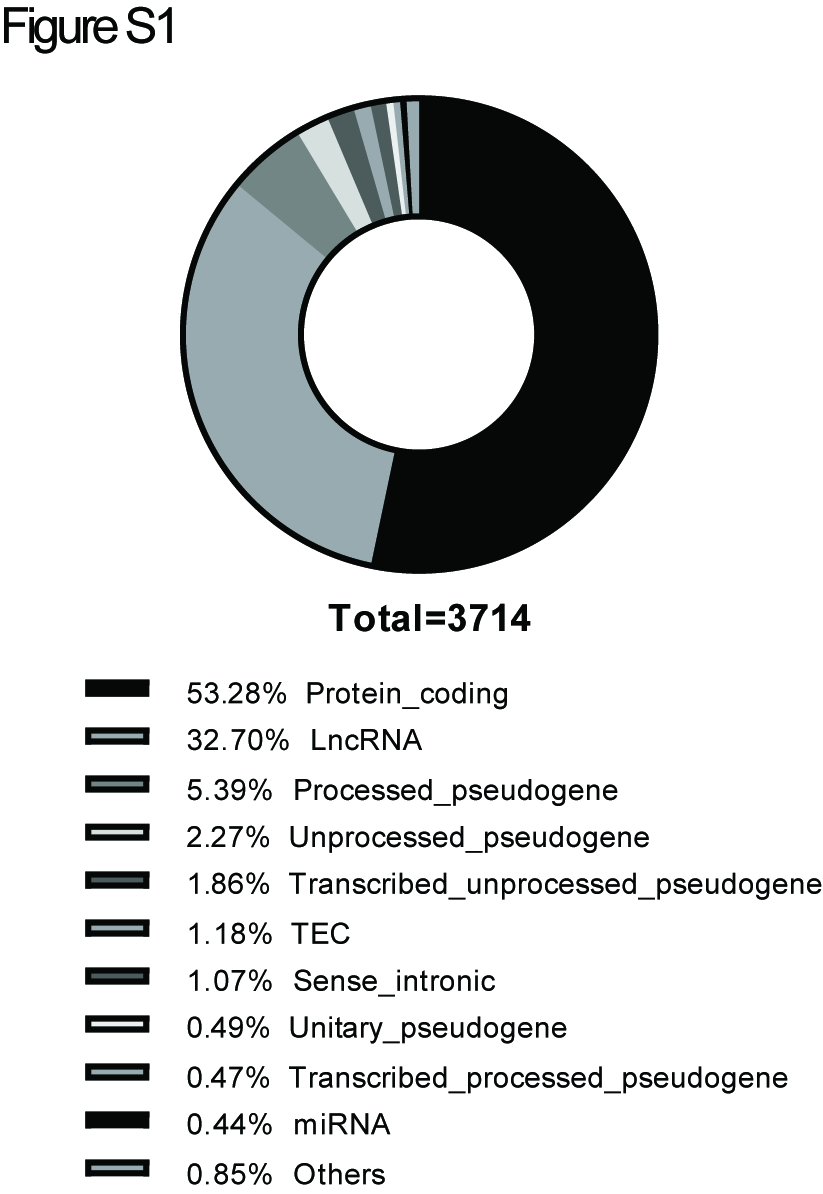

Supplement: Supplementary Figure 1 — Pie chart for the composition of differentially expressed genes (DEGs). The DEGs consisted of 53.28% protein-coding genes, 32.70% lncRNAs, 0.44% miRNAs, etc. [file Image_1.tif]

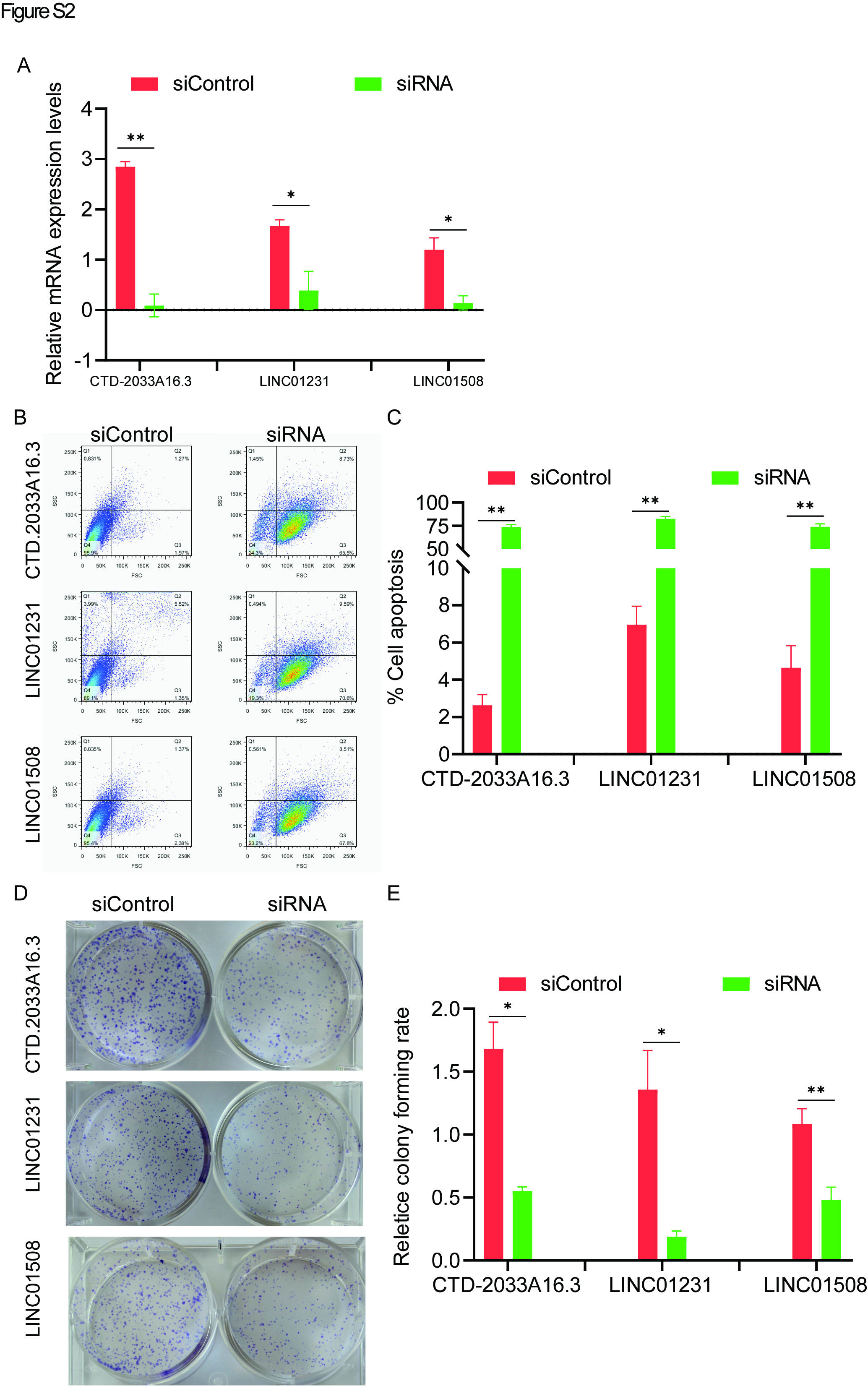

Supplement: Supplementary Figure 2 — Effects of CTD-2033A16.3, LINC01231, and LINC01508 on the apoptosis and proliferation of hepatocellular carcinoma (HCC) cells HUH-7. (A) qPCR analysis of lncRNA expression after transfection with the lncRNA-targeted siRNAs. (B, C) The inhibition of CTD-2033A16.3, LINC01231, and LINC01508 by siRNAs leads to the promotion of cell apoptosis rate. (D, E) The inhibition of CTD-2033A16.3, LINC01231, and LINC01508 by siRNAs leads to the inhibition of the cell colony forming rate. Values are displayed as mean ± SD for three independent replicates. *p < 0.05, **p < 0.01. [file Image_2.tif]

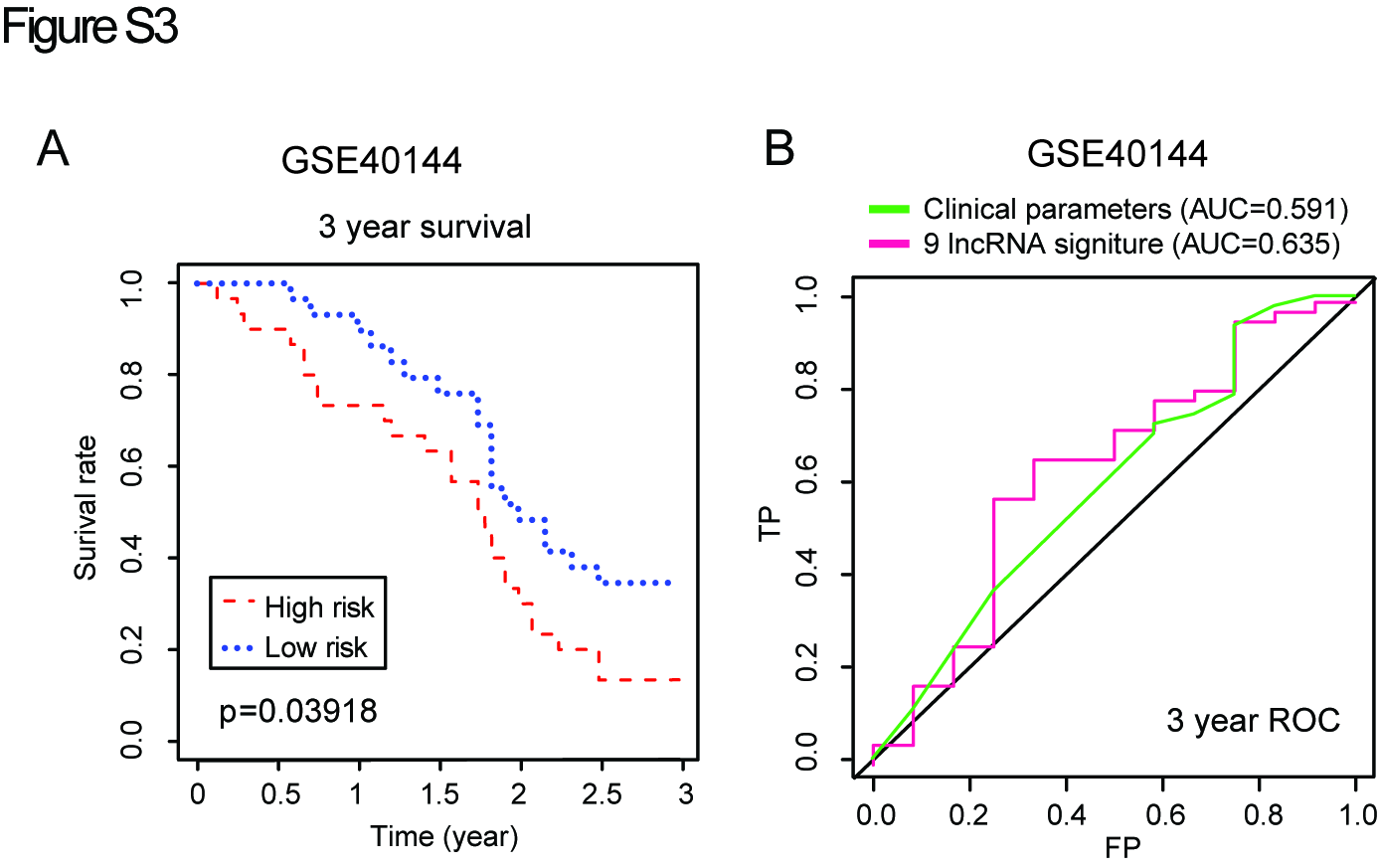

Supplement: Supplementary Figure 3 — Prognostic value of ferroptosis-associated lncRNA signature in GSE40144. (A) Kaplan–Meier analysis was used to verify the prognostic values of novel lncRNA-based signature in hepatocellular carcinoma patients from GSE40144. (B) Time-dependent receiver operating characteristic curve for the survival prediction of the risk score model in the GSE40144 cohort. [file Image_3.tif]

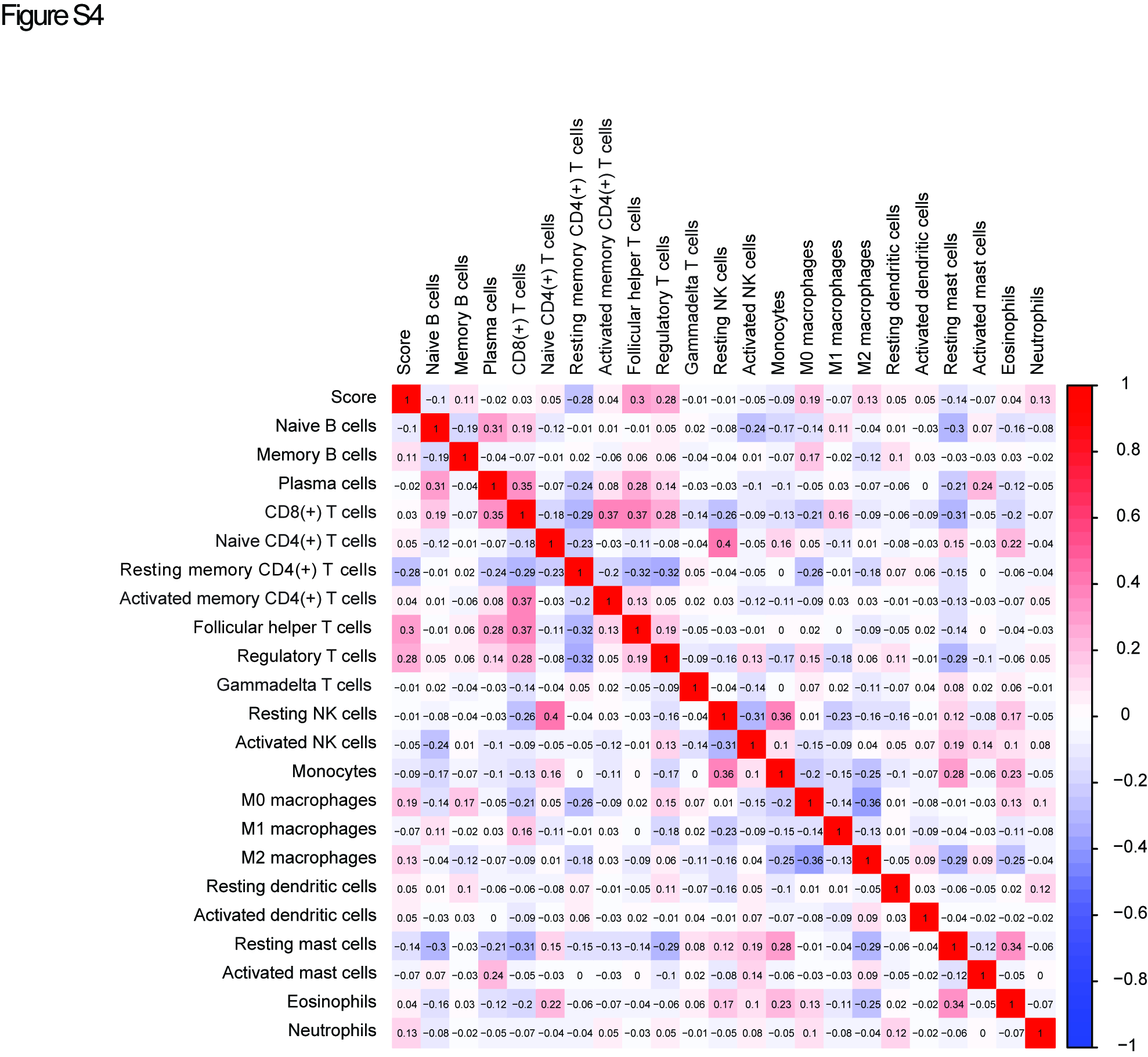

Supplement: Supplementary Figure 4 — Spearman’s correlation on the association between the risk score of the signature and immune cells. [file Image_4.tif]
