# Supplementary material for: Construction of a Ferroptosis-Related Nine-lncRNA Signature for Predicting Prognosis and Immune Response in Hepatocellular Carcinoma
Source: Front Immunol. 2021 Sep 17;12:719175. doi: 10.3389/fimmu.2021.719175 (PMC8484522; doi:10.3389/fimmu.2021.719175)
Supplement: Supplementary file 6 [file Table_2.docx]

**Supplementary Table S2. The sequences used in this study**

| Primers |  |  |
| --- | --- | --- |
| Gene | Forward primer | Reverse primer |
| CTD.2033A16.3 | GTTGCTCCGAAATGGGTGCC | CTCTGGCTCCACGCCATAAA |
| CTD.2116N20.1 | GGTCAGTGATTTCTTAACTGCCA | AGCTTTTACTTGAACCTGCTGT |
| CTD.2510F5.4 | ACCCTACTTCGGAGACCACT | CCCAACACCCATCTGACCTG |
| DDX11.AS1 | CGGGAACTGGGATCTCAACC | AGTACACCTGGCGCTAATCG |
| LINC00942 | CCTGGATTGTGGGCCTTGAA | TACTGTTTCCCGCAGACACC |
| LINC01224 | ACGTGCACAGACAGCTAAGA | ATCATCCACGGGAGTGACGA |
| LINC01231 | ACCTAGGGCTTCCTTCGAGT | TGGCAGAACCTAAGCTGGTG |
| LINC01508 | GGATGCATCTGCAGCTTGTG | TGAGCCATAGCACACGGAAG |
| ZFPM2.AS1 | CCCAGGGAGAGTATGGAGTGA | AGTTGCAAGATGACGCTCAGT |
| 18srRNA | AGGCCCTGTAATTGGAATGAGTC | GCTCCCAAGATCCAACTACGAG |
| siRNA |  |  |
| Gene | Sense | Antisense |
| CTD.2033A16.3 | GCUCCGUGGAGACGAAUUUTT | AAAUUCGUCUCCACGGAGCTT |
| LINC01231 | GCUCAAAGUGCAUUCCAUUTT | AAUGGAAUGCACUUUGAGCTT |
| LINC01508 | GCACCUACACGUGUCACUUTT | AAGUGACACGUGUAGGUGCTT |
